# Effect of fed- versus fasted state resistance training during Ramadan on body composition and selected metabolic parameters in bodybuilders

**DOI:** 10.1186/1550-2783-10-23

**Published:** 2013-04-25

**Authors:** Khaled Trabelsi, Stephen R Stannard, Zohra Ghlissi, Ronald J Maughan, Choumous Kallel, Kamel Jamoussi, Khaled M Zeghal, Ahmed Hakim

**Affiliations:** 1 University of Sfax, Laboratory of Pharmacology, Faculty of Medicine, Sfax, 3029, Tunisia; 2 School of Sport and Exercise, Massey University, Palmerston North, New Zealand; 3 School of Sport and Exercise Sciences, Loughborough University, Loughborough, UK; 4 Laboratory of Hematology, CHU Habib Bourguiba, Sfax, Tunisia; 5 Department of Biochemistry of the Hedi Chaker University Hospital, Sfax, 3029, Tunisia

**Keywords:** Resistance training, Dehydration, Renal function, Body fat percentage, Islamic fasting

## Abstract

**Background:**

Muslim bodybuilders often continue training during Ramadan. However, the effect of resistance training in a fasted versus a fed state during Ramadan on body composition and metabolic parameters in bodybuilders is not well known. The aim of this study was to evaluate the effects of resistance training in a fasted versus a fed state during Ramadan on body composition and metabolic parameters in bodybuilders.

**Methods:**

Sixteen men were allocated to two groups: Eight practicing resistance training in the late afternoon in a fasted state (FAST), and eight training in the late evening in an acutely fed state (FED) during Ramadan. All visited the laboratory in the morning two days before the start of Ramadan (Bef-R) and on the 29th day of Ramadan (End-R) for anthropometric measurement, completion of a dietary questionnaire, and provision of fasting blood and urine samples.

**Results:**

Body mass and body fat percentage remained unchanged in FAST and FED during the whole period of the investigation. Both FAST and FED experienced an increase in the following parameters from Bef-R to End-R: urine specific gravity (1%; p = 0.028, p = 0.004 respectively), serum concentrations of urea (4%, p *=* 0.006; 7%, p = 0.004 respectively), creatinine (5%, p *=* 0.015; 6%, p = 0.04 respectively), uric acid (17%; p < 0.001, p = 0.04 respectively), sodium (1%; p = 0.029, p = 0.019 respectively), chloride (2%; p = 0.039, p = 0.004 respectively), and high-density lipoprotein cholesterol (11%, p *=* 0.04; 10%, p = 0.04 respectively).

**Conclusion:**

Hypertrophic training in a fasted or in a fed state during Ramadan does not affect body mass and body composition of bodybuilders. Additionally, Ramadan fasting induced changes in urinary and some biochemical parameters, but these changes were not different according to when the training occurred.

## Introduction

Most Muslims fast during the holy month of Ramadan from dawn till sunset, when they neither eat nor drink, as it forms one of the fundamental obligations of the Muslim faith [1]. The Ramadan month occurs eleven days earlier every year and thus over time may occur in any of the four seasons [2]. Therefore, the length of the daily fast during Ramadan varies from 11–18 hours in tropical countries [3].

Not only is the eating pattern by necessity altered during Ramadan, the type of food eaten during the night may also be different from that usually consumed during the rest of the year [4]. Energy and water intake are often reduced during this month [5,6], which may result in reduced body mass [5,6] and changed hydration status.

Participants of Ramadan often maintain physical activity during the holy month for recreation and health purposes, and this has the potential to further affect body mass and produce dehydration. The few investigations that have examined the effect of Ramadan fasting on the hydration status of sportsmen report conflicting findings. For example, while urine osmolarity increased in Emirates soccer players [7] indicating a state of dehydration, the absence of change in urine specific gravity has been reported in Turkish [8] and Tunisian [9] soccer players. Further, the interaction between participation in Ramadan and exercise and subsequent effects on circulating metabolites are also poorly understood. Resting serum glucose has been reported to decrease during Ramadan in moderately trained runners [10], soccer and basketball players [11] and runners [12], but not to change in elite rugby players [5], weight lifters [13] and physically active men [1,2]. Part of this conflict in findings may be due to the difference in time of the day, during which the training was conducted. For example, if the training was performed in the afternoon or early evening towards the latter part of the daily fast, the physiological stresses would be quite different to those if training was undertaken soon after breaking the fast. Certainly it is now well established that training after a 12 hour fast induces significantly different metabolic adaptations than training performed immediately after a meal [13].

Muslim athletes, including strength athletes, employ a variety of coping strategies to deal with the challenges of training and/or competing during the month of Ramadan [14,15]. Some Muslim athletes train at night to prevent dehydration, hypoglycemia and possible decrements in performance. However, it has been demonstrated that resistance training (weight lifting) in a fasted state affects the post-workout anabolic response to weight training more favorably than training after a fed-state, but only when a carbohydrate/protein/leucine mixture was ingested following a heavy resistance training session [16]. There is an ample amount of evidence that ingestion of protein after exercise will stimulate net muscle protein synthesis [17]. This begs the question as to whether the daytime resistance training during Ramadan (i.e. fasted state training), might accelerate adaptations to training and ultimately result in increasing muscle mass, although risk of dehydration and hypoglycemia may be increased.

Published data describing the effects of Ramadan on body composition and biochemical parameters following resistance training are scarce. The only published studies that have observed the effects of resistance exercise during Ramadan have lacked the control group performing equivalent exercises in the acutely fasted state [18,19], therefore, no specific effects of resistance training while fasted were identified.

It is clear that well designed scientific studies, investigating the effect of resistance training in the fasted state during Ramadan on body composition and markers of renal function, inflammation and immunity, are currently lacking. The aim of this study was to evaluate the effects of resistance training during Ramadan on body composition and markers of renal function, inflammation and immunity of bodybuilders as well as to ascertain whether there is a difference between daytime resistance training in a fasted state and nighttime resistance training in a fed state. We hypothesized that resistance training could be safely practiced during Ramadan with decrements in body composition and circulating markers of health (renal function, immunity and inflammation). It was also hypothesized that resistance training in the fasted state would lead to increased levels of markers of dehydration, while positively affecting the change in lean body mass when compared to nighttime training after the fast was broken.

## Methods

### Subjects

Sixteen male bodybuilders were recruited into the study and randomly allocated to two groups: Eight participants trained in a fasted state (FAST), and 8 trained in a fed state (FED) during Ramadan. Each of the subjects regularly performed bodybuilding (hypertrophic program) for recreational purposes at least 3 times/week but did not participate in national or international bodybuilding competitions. The subjects’ descriptive characteristics are provided in Table 1.

**Table 1:** Descriptive characteristics, M ± SD

	**FAST**	**FED**
Age (y)	25 ± 3	25 ± 2
Mass (kg)	79.9 ± 5.5	79.1 ± 3.2
Height (cm)	176 ± 3	174 ± 5
BMI (kg · m^-2^)	25.8 ± 0.4	26.0 ± 1.7
BF%	15 ± 2	14 ± 1
LBM (kg)	68.2 ± 3.5	68.3 ± 2.6
Years of resistance training	1.6 ± 0.6	1.5 ± 0.5
Number of training session/week	3.8 ± 0.5	3.6 ± 0.7
Back squat 10 RM (Kg)	98.7 ± 25.3	104.4 ± 26.4
Bench press 10 RM (Kg)	63.7 ± 11.3	60.1 ± 8.1
Barbell row 10 RM (Kg)	50.1 ± 8.9	55 ± 8.9
Seated shoulder press behind the neck 10 RM (Kg)	44.4 ± 5.6	46.2 ± 9.2
Biceps curl 10 RM (Kg)	30.6 ± 4.9	35 ± 5.3
Lying triceps curl 10 RM (Kg)	30.6 ± 4.2	33.7 ± 3.5

Note: FAST = subjects training in a fasted state; FED = subjects training in a fed state. BMI = body mass index; BF% = body fat percentage; LBM = lean body mass; RM = repetition maximum.

To qualify as subjects the men a) were nonsmokers b) had no current or past history of anabolic steroid use (according to self-report); c) had at least 1 year of bodybuilding training experience; d) had not ingested any ergogenic supplement for an 8-week period prior to the start of the study; and e) agreed not to ingest any other nutritional supplements, or non-prescription drugs that might affect the study parameters.

Prior to enrolling in the study, subjects were informed of the experimental procedures as well as the potential risks and benefits associated with the study; however, subjects were not informed of the study’s purpose. To be included in the study, each subject provided written consent in accordance with the Declaration of Helsinki. The study was approved by the research ethics committee of the Faculty of Medicine of the University of Sfax, Sfax, Tunisia.

### Experimental design

Ramadan began on August 1 and ended on August 30, 2011. The average duration of the fast was approximately 15 h. The study was conducted in Tunisia, where daytime temperatures were 34 ± 1°C and relative humidity was 57 ± 4%. Subjects visited the laboratory on two separate occasions: two days before Ramadan (Bef-R) and on the 29th day of Ramadan (End-R). In the morning of each visit (approximately 10:30 a.m.), they underwent anthropometric measurements, completed a dietary questionnaire, and provided fasting blood and urine samples. They were instructed not to consume any food or energy-containing beverage after 11:00 p.m. on the day before each visit. Because of the time of sunset, this meant that the fasting subjects had only four hours (between 7:00 and 11:00 p.m.) on the evening before the test at End-R in which to consume food and fluid. Seventeen days before the beginning of Ramadan, subjects underwent a test of 10 repetitions maximum (10 RM) for the following exercises: bench press, barbell squat, biceps curl, lying triceps curl, seated shoulder press behind the neck and barbell row. During the 10 RM testing, the mass of all weight plates and bars that were used was determined with a precision scale. The actual mass of all plates and bars was then used to calculate the 10 RM of each exercise. During the 10 RM tests, each subject had a maximum of 5 attempts on each exercise with 2- to 5-minute intervals between attempts. After each attempt, subjects add or remove weight as required. After the 10 RM load in a specific exercise was determined, an interval no shorter than 10 minutes was allowed before the 10 RM determination of the next exercise. Standard exercise techniques were followed for each exercise. No pause was allowed between the eccentric and the concentric phase of a repetition or between repetitions. For a repetition to be successful, a complete range of motion as is normally defined for the exercise had to be completed. The testing procedures met the criteria proposed by Kraemer and Fry [20].

To avoid potential confounding effects of prior exercise on blood circulating biochemical and hematological parameters, subjects were instructed to practice only a light training session within the 36-h period before they undertook the laboratory assessments.

During the two weeks before and during Ramadan, subjects recorded their exercise sessions along with their rating of perceived exertion (RPE) on the Borg scale [21] (Table 2) in a training journal. All subjects were familiarized with the use of the RPE scale before the commencement of the study. During Ramadan, exercise sessions of FAST occurred in the late afternoon (between 4:00 and 6:00 p.m.) and those of FED occurred at night (between 9:00 and 10:00 p.m.) after the break of fasting. The number of training sessions, sets, repetitions in each set, total training volume and RPE did not change in either FAST or FED during the duration of the study (Table 2). Additionally, no differences in the number of training sessions, number of sets, the number of repetition in each set, total training volume and RPE existed between FAST and FED at any time period.

**Table 2:** Training data before and during Ramadan, M ± SD

	**Before Ramadan**		**During Ramadan**	
	**FAST**	**FED**	**FAST**	**FED**
Number of training session/week	3.8 ± 0.5	3.7 ± 0.6	3.6 ± 0.4	3.6 ± 0.5
Number of sets /training session	20 ± 1	20 ± 1	20 ± 1	20 ± 1
Number of repetition/sets	9.68 ± 0.76	9.42 ± 0.69	9.37 ± 0.92	9.78 ± 0.87
Total training volume	4047 ± 463	3940 ± 373	3914 ± 440	4091 ± 498
RPE	8 ± 1	8 ± 1	8 ± 1	8 ± 1

Note: FAST = subjects training in a fasted state; FED = subjects training in a fed state. RPE = rating of perceived exertion.

### Bodybuilding training program

The resistance training program employed both free weights and machines. The primary goal of the program was to increase muscle mass (hypertrophic program), so closely followed the principles documented by the American College of Sports Medicine (ACSM) for producing effective gains in muscle hypertrophy [22]. Briefly, four training sessions each week were conducted by each subject, and each training session was composed of four to six specific exercises. Each exercise was performed in four sets with a load of 10 RM and intervals of 2–3 min between sets. The exercises were conducted first with the major muscle groups and, then, with the smaller muscle groups. Training intensity was increased progressively as needed, by adding weight lifted, to ensure that target intensity was maintained as subjects got stronger and set workloads became easier. The first day of the week’s training program was devoted to the development of quadriceps, hamstring and calves using barbell squat, hack squat, leg extensions, lying leg curl and seated calve raise. The second day was devoted to the development of back and triceps using barbell row, one-arm dumbbell row, wide-grip lat pulldown, dip machine, lying triceps curl and standing dumbell triceps extension, and the third devoted to the development of shoulders using seated shoulder press behind the neck, side lateral raise, front dumbbell raise and seated bent-over rear deltoid raise. The fourth day was devoted to the development of chest and biceps using barbell bench press (medium grip), barbell incline bench press (medium grip), decline barbell bench press, barbell curl, one arm dumbbell preacher curl and hammer curls. Other exercises were incorporated in the training program each week. A certified strength and conditioning specialists closely supervised all subjects perform each training session. The total training volume was estimated using the following equation: training volume = total number of sets × total number of repetitions [22].

### Body composition

Body weight was measured to the nearest 10 g using a calibrated electronic scale (Seca Instruments Ltd., Germany), and height was measured to the nearest 5 mm using a stadiometer. Body mass index (BMI) was then calculated. Skinfold thickness was measured by an experienced (trained) anthropometrist in triplicate using calibrated Harpenden calipers (Harpenden, UK) at four standardized sites (biceps, triceps, subscapular, and suprailium). Those measurements followed the protocol of the International Society for the Advancement of Kinanthropometry [23]. The level of technical error measurements of the anthropometrist was 6%.

Body fat percentage (BF%) was estimated from skinfold measures using a previously published algorithm [24]. Lean body mass (LBM) was calculated as body weight minus body fat mass.

### Dietary intake analysis

Subjects were instructed to record the estimated quantities of all food and beverages consumed during the week before Ramadan and then three days/week during Ramadan. Dietary records were analyzed using the Bilnut program (Nutrisoft, Cerelles, France) and the food-composition tables of the National Institute of Statistics of Tunis (1978). Total water intake was defined as the fluid volume of consumed beverages plus the water content of consumed foods.

### Urine specific gravity

Urine specific gravity was assessed from 30 ml of urine collected from each subject immediately before the anthropometrical measurement. It was measured to the nearest 0.001 unit with a hand refractometer (Atago,Japan).

### Serum biochemistry

During each session, venous blood samples (~7 ml) were taken from an antecubital vein and collected into a plain blood tube in a seated position in a room controlled temperature and relative humidity (23 ± 3°C and 47% ± 5% respectively). An aliquot of blood was immediately removed and mixed with ethylene diaminetetraaceticacid (EDTA) as an anticoagulant. These blood samples were analyzed for total leucocytes, neutrophils, lymphocytes and monocytes using an automated analyzer (Beckman coulter, Coulter LH 750 Analyzer, UK) according to the manufacturer’s protocol. The remaining blood was allowed to clot and was then centrifuged at 1500 *g* for 10 min at 4°C. An aliquot of the serum was used to measure serum glucose immediately after the centrifugation step; the remainder was then stored at −20°C for subsequent analysis. An automated analyzer (Beckman Coulter DXC 600, UK) measured the concentrations of biochemical parameters using the appropriate reagents (Beckman Coulter, UK). Glucose, uric acid, total cholesterol (TC) and triglycerides (TG) were determined using an enzymatic colorimetric method (glucose oxidase, uricase, lipoprotein lipase-glycerol kinase reactions, cholesterol esterase-cholesteroloxidase reactions, respectively). Urea was determined using an enzymatic method. Urea is first converted by urease into ammonia which is then estimated by the reaction with α-ketoglutarate catalyzed by glutamic dehydrogenase. Creatinine concentrations were determined by the Jaffé method in which creatinine directly reacts with alkaline picrate resulting in the formation of a red colour. Creatinine clearance was determined using the formula of Cockroft and Gault. [25]: Creatinine clearance (ml•min^-1^) = 1.25 × body mass (kg) × (140 - age (y)): creatinine (μmol•l^-1^). Sodium, potassium and chloride concentrations were determined by potentiometry. C-reactive protein concentrations were determined using a turbidimetric method. In the reaction, C-reactive protein combines with specific antibody to form insoluble antigen-antibody complexes. High-density lipoprotein cholesterol (HDL-C) concentrations were determined by immuno-inhibition. Low-density lipoprotein cholesterol (LDL-C) was calculated using the Friedewald formula [26]: LDL-C (mmol•l^-1^) = TC – HDL-C – TG: 2.2. The ratios TC: HDL-C and LDL-C: HDL-C were derived from the respective concentrations. Creatine kinase (CK), lactatedehydrogenase (LDH), alanine aminotransferase (ALT), aspartate aminotransferase (AST), alkaline phosphatase (AP) and γ-glutamyl transferase (γ-GT) activity were determined using an enzymatic method.

### Statistical analyses

All statistical tests were performed using STATISTICA Software (StatSoft, Paris, France). The distribution of all dependent variables was examined by the Shapiro-Wilk test and was found not to differ significantly from normal. A 2 (periods) × 2 (FAST or FED) repeated-measures analysis of variance (ANOVA) was applied. If a significant interaction was present, a Bonferroni post-hoc test was performed where appropriate. If a non-significant interaction was present, a paired or independent *t*-test was preformed where appropriate.

Effect sizes were calculated as partial eta-squared η_*p*_^2^ to estimate the meaningfulness of significant findings. Partial eta squared values of 0.01, 0.06 and 0.13 represent small, moderate, and large effect sizes, respectively. Statistical significance was set at *P* < 0.05. All data are expressed as mean ± standard deviation (*M* ± *SD*).

## Results

### Dietary intake

Dietary intake before and during Ramadan is presented in Table 3. Estimated mean daily energy intake Bef-R was similar between FAST and FED. Calculated daily energy intake during Ramadan did not significantly change in either group compared with Bef-R. Carbohydrate and fat consumption increased by 9% (p = 0.003) and 5% (p = 0.05) respectively in FED during Ramadan, though consumption of these macronutrients did not significantly change in FAST during the month. Protein consumption during Ramadan did not change in either group compared with Bef-R. Expressed as a percentage of daily macronutrient intake, protein, carbohydrates, and fat consumption did not change in FAST and FED during Ramadan. Further, the proportion of total energy expressed as grams per kilogram body mass per day from carbohydrates increased in FED (p = 0.006); and remained unchanged in FAST during Ramadan. Both fat and protein intakes (expressed as grams per kilogram body mass per day) did not change during Ramadan in either group. Potassium intake in FED decreased by 14% (p = 0.019) from Bef-R to End-R, and it remained unchanged in FAST. Total water intake decreased by 15% (p = 0.039) in FAST and by 13% (p = 0.004) in FED during Ramadan.

**Table 3:** Dietary intake before and during Ramadan, M ± SD

	**Before Ramadan**		**During Ramadan**	
	**FAST**	**FED**	**FAST**	**FED**
Energy intake (kcal · d^-1^)	3492 ± 253	3409 ± 209	3434 ± 266	3613 ± 245
Protein (g · d^-1^)	125 ± 10	133 ± 8	127 ± 9	129 ± 6
Protein (%)	14 ± 1	16 ± 1	15 ± 1	14 ± 1
Protein (g.Kg.d^-1^)	1.6 ± 0.1	1.7 ± 0.1	1.6 ± 0.1	1.6 ± 0.1
Fat (g · d^-1^)	105 ± 8	101 ± 7	104 ± 7	106 ± 6*
Fat (g.Kg.d^-1^)	1.3 ± 0.2	1.3 ± 0.1	1.3 ± 0.1	1.3 ± 0.1
Fats (%)	27 ± 4	27 ± 2	27 ± 3	26 ± 2
Carbohydrate (g · d^-1^)	511 ± 72	492 ± 44	497 ± 64	536 ± 55**
Carbohydrate (g.kg.d^-1^)	6.4 ± 0.8	6.2 ± 0.5	6.3 ± 0.6	6.8 ± 0.6**
Carbohydrate (%)	58 ± 5	58 ± 3	58 ± 4	59 ± 2
Potassium (g . d^-1^)	2.5 ± 0.4	2.8 ± 0.4	2.4 ± 0.4	2.4 ± 0.3*
Sodium (g . d^-1^)	6.9 ± 1.1	6.8 ± 1.1	7 ± 1	6.9 ± 1
Total water intake (L · d^-1^)	4.5 ± 0.4	4.5 ± 0.5	3.8 ± 0.7*	3.9 ± 0.4**

Significantly different from before Ramadan: * (*P* < 0.05); ** (*P* < 0.01). Note: FAST = subjects training in a fasted state; FED = subjects training in a fed state.

### Body composition

Body mass and body composition before and at the end of Ramadan are shown in Table 4. The two-way ANOVA (Ramadan × group) for body mass, BF% and LBM showed no significant effects for Ramadan, no significant effect for group and no significant effect for Ramadan × group interaction. Paired samples *t*-test revealed that body mass, BF% and LBM did not change during the duration of the study in FAST nor FED. Independent samples *t*-test showed no significant differences in these parameters between the two groups at any time period.

**Table 4:** Body mass and body composition before and at the end of Ramadan, M ± SD

**Group**				**Ramadan effect**			**Group effect**			**Ramadan × group effect**		
				***F(1,14)***	***P*-value**	**η**_***p***_^**2**^	***F(1,14)***	***P*-value**	**η**_***p***_^**2**^	***F(1,14)***	***P*-value**	**η**_***p***_^**2**^
Body mass (kg)	FAST	79.9 ± 5.5	79.2 ± 4.6	1.06	0.32	0.07	0.043	0.83	0.003	0.72	0.41	0.05
	FED	79.1 ± 3.2	79 ± 3.7									
BF%	FAST	14.6 ± 2.1	13.9 ± 1.9	10.92	0.005	0.043	1.21	0.29	0.08	0.85	0.37	0.05
	FED	13.6 ± 1.3	13.2 ± 1									
LBM (kg)	FAST	68.2 ± 3.5	68 ± 3.1	0.023	0.88	0.01	0.062	0.81	0.004	0.31	0.59	0.02
	FED	68.3 ± 2.6	68.6 ± 2.9									

Note: FAST = subjects training in a fasted state; FED = subjects training in a fed state. BF% = Body fat percentage; LBM = lean body mass; η_*p*_^2^ = effect sizes. Before Ramadan (Bef-R) = 2 days before beginning the fast; end of Ramadan (End-R) = 29 days after beginning the fast.

### Urine specific gravity

There was a significant effect for Ramadan (F_(1,14)_ = 20.1; p < 0.001; η_*p*_^2^ =0.6), no significant effect for group (F_(1,14)_ = 1; p = 0.33; η_*p*_^2^ =0.06) and no significant Ramadan × group interaction (F_(1,14)_ = 0; p = 0.77; η_*p*_^2^ =0.006 ) on urine specific gravity. Paired samples *t*-test showed urine specific gravity in FAST increased significantly (*p* = 0.028) from 1.019 ± 0.007 at Bef-R to 1.029 ± 0.005 at End-R. Similarly, urine specific gravity in FED increased significantly (p = 0.004) from 1.018 ± 0.004 at Bef-R to 1.027 ± 0.004 at End-R. Independent samples *t*-test revealed that there was no difference in urine specific gravity values between FAST and FED at each time period.

### Renal-function markers

Renal function markers before and at the end of Ramadan are presented in Table 5. Though the two-way ANOVA (Ramadan × group) for urea, creatinine, creatinine clearance and uric acid revealed a significant effect for Ramadan, there was no significant group effect or Ramadan × group interaction. Paired samples *t*-test showed a significant increase of urea in FAST by 4% (p = 0.006) and by 7% (p = 0.031) in FED from Bef-R to End-R. Similarly, creatinine values at End-R increased by 5% in FAST (p *=* 0.015) and by 6% in FED (p = 0.04). However, creatinine clearance did not change throughout the study in either group. For uric acid concentrations, paired samples *t*-test showed a significant increase by 17% in FAST and FED (p < 0.001, p = 0.04 respectively) from Bef-R to End-R. Independent samples *t*-test revealed no significant differences on these parameters between the two groups at any time period.

**Table 5:** Renal function markers and serum electrolyte concentrations before and at the end of Ramadan, M ± SD

**Group**				**Ramadan effect**			**Group effect**			**Ramadan × group effect**		
				***F(1,14)***	***P*-value**	**η**_***p***_^**2**^	***F(1,14)***	***P*-value**	**η**_***p***_^**2**^	***F(1,14)***	***P*-value**	**η**_***p***_^**2**^
Urea (mmol•l^-1^)	FAST	4.55 ± 0.33	4.72 ± 0.39**	15.05	0.002	0.52	0.06	0.81	0.004	1.35	0.26	0.08
[CV = 5.7%]^a^	FED	4.43 ± 0.18	4.76 ± 0.19*									
Creatinine (μmol•l^-1^)	FAST	89.87 ± 3.18	94.12 ± 4.26*	15	0.002	0.51	1.17	0.3	0.07	0.1	0.76	0.01
[CV = 3%]	FED	87.32 ± 5.32	92.62 ± 3.78*									
Uric acid (μmol•l^-1^)	FAST	309.75 ± 68.96	356.75 ± 63.86***	22.4	<0.001	0.61	1.21	0.28	0.08	0	0.99	0
[CV = 2.8%]	FED	279 ± 56.07	326.12 ± 44.73*									
Creatinine clearance	FAST	129.27 ± 9.02	125.09 ± 11.97	5.36	0.04	0.27	0.008	0.93	0.0005	0.19	0.67	0.01
(ml•min-1)	FED	130.61 ± 6.86	124.46 ± 7.96									
Sodium (mmol•l^-1^)	FAST	142.25 ± 2.71	144.25 ± 1.16*	17.9	<0.001	0.56	0.2	0.64	0.01	0	1	0
[CV = 2.7%]	FED	142.62 ± 1.41	144.62 ± 1.68*									
Potassium (mmol•l^-1^)	FAST	4.49 ± 0.42	4.74 ± 0.55*	3.09	0.1	0.18	0.02	0.9	0.001	10.66	0.006	0.43
[CV = 2.8%]	FED	4.67 ± 0.37	4.6 ± 0.23									
Chloride (mmol•l^-1^)	FAST	102.37 ± 1.68	104.25 ± 1.83*	20.55	<0.001	0.6	0.89	0.36	0.05	0.17	0.68	0.01
[CV = 2.9%]	FED	101.5 ± 1.19	103.75 ± 2.05**									

Significantly different from before Ramadan: * (*P* < 0.05); ** (*P* < 0.01); *** (*P* < 0.001). Note: FAST = subjects training in a fasted state; FED = subjects training in a fed state; ^a^ = inter-assay coefficient of variance. Before Ramadan (Bef-R) = 2 days before beginning the fast; end of Ramadan (End-R) = 29 days after beginning the fast.

### Serum electrolytes

Serum electrolytes concentrations before and at the end of Ramadan are shown in Table 5. For serum sodium and chloride concentrations, there was a significant effect for Ramadan, no significant effect for group and no significant Ramadan × group interaction. Paired samples *t*-test showed a significant increase by 1% in FAST and FED for serum sodium concentrations (p = 0.029, p = 0.019 respectively) and by 4% in FAST and FED for serum chloride concentrations (p = 0.039, p = 0.004 respectively) from Bef-R to End-R. Independent samples *t*-test showed no significant differences in these parameters between the two groups at any time period.

There was a significant effect for Ramadan, no significant effect for group and a significant Ramadan × group interaction for serum potassium concentrations. The post hoc test showed a significant increase by 6% from Bef-R to End-R (p = 0.019). However, serum potassium concentrations of FED remained unchanged over the whole period of the investigation. No differences were found in potassium values between FAST and FED at any time period of the investigation.

### Serum lipid and glucose

Serum lipid and glucose concentrations before and at the end of Ramadan are summarized in Table 6. The two-way ANOVA (Ramadan × group) for TG and TC and LDL-C concentrations showed no significant effects for Ramadan, no significant effect for group or the interaction between the two. Paired samples *t*-test revealed that TG and TC concentrations did not change during the duration of the study in either group. Independent samples *t*-test showed no significant differences in these parameters between the two groups at any time period.

**Table 6:** Serum lipid and glucose concentrations before and at the end of Ramadan, M ± SD

**Group**				**Ramadan effect**			**Group effect**			**Ramadan × group effect**		
				***F(1,14)***	***P*-value**	**η**_***p***_^**2**^	***F(1,14)***	***P*-value**	**η**_***p***_^**2**^	***F(1,14)***	***P*-value**	**η**_***p***_^**2**^
TG (mmol•l^-1^)	FAST	0.73 ± 0.16	0.75 ± 0.15	1.37	0.26	0.08	0.02	0.89	0.001	0.29	0.59	0.02
[CV = 2.7%] ^a^	FED	0.74 ± 0.11	0.75 ± 0.11									
TC (mmol•l^-1^)	FAST	3.82 ± 0.34	3.87 ± 0.35	0.006	0.94	0	0.45	0.51	0.03	0.023	0.2	0.11
[CV = 3%]	FED	3.98 ± 0.34	3.93 ± 0.35									
HDL-C (mmol•l^-1^)	FAST	1.11 ± 0.26	1.24 ± 0.20*	23.87	<0.001	0.62	0.1	0.75	0.01	0.02	0.9	0.01
[CV = 3.1%]	FED	1.15 ± 0.16	1.26 ± 0.18*									
LDL-C (mmol•l^-1^)	FAST	2.37 ± 0.3	2.29 ± 0.26	0.05	0.82	0.003	1.92	0.19	0.12	0.07	0.08	0.19
	FED	2.49 ± 0.37	2.6 ± 0.38									
TC: HDL-C	FAST	3.58 ± 0.82	3.18 ± 0.44	17.52	<0.001	0.55	0.02	0.89	0	0.02	0.9	0.001
	FED	3.53 ± 0.59	3.15 ± 0.43									
LDL-C: HDL-C	FAST	2.44 ± 0.79	2.05 ± 0.43	9.06	0.009	0.39	0.08	0.78	0.01	1.9	0.19	0.11
	FED	2.39 ± 0.57	2.34 ± 0.41									
Glucose (mmol•l^-1^)	FAST	4.97 ± 0.53	4.88 ± 0.58	1.71	0.21	0.1	0.78	0.39	0.05	0.044	0.83	0.03
[CV = 2.1%]	FED	4.77 ± 0.37	4.66 ± 0.47									

Significantly different from before Ramadan: * (*P* < 0.05). Note: FAST = subjects training in a fasted state; FED = subjects training in a fed state; ^a^ = inter-assay coefficient of variance. TG = triglycerides; TC = total cholesterol; HDL-C = high-density lipoprotein cholesterol; LDL-C = low-density lipoprotein cholesterol. Before Ramadan (Bef-R) = 2 days before beginning the fast; end of Ramadan (End-R) = 29 days after beginning the fast.

There was a significant effect for Ramadan, no significant effect for groups and a significant Ramadan × group interaction on HDL-C concentrations. Paired samples *t*-test showed a significant increase in FAST and FED by 11% (p = 0.04, p = 0.04 respectively) from Bef-R to End-R. Independent samples *t*-test revealed that there was no difference in HDL-C values between FAST and FED at each time period.

For TC: HDL-C and LDL-C: HDL-C ratios, there was a significant effect for Ramadan, no significant effect for group and no significant Ramadan × group interaction. Paired samples *t*-test showed that TC: HDL-C and LDL-C: HDL-C did not change throughout the study in FAST nor FED. No differences were found in TC: HDL-C and LDL-C: HDL-C ratios between FAST and FED at any time period of the investigation.

There was no significant effect for Ramadan, no significant effect for group or interaction between the two on serum glucose concentrations. Paired samples *t*-test showed that glucose concentrations did not change throughout the study in FAST nor FED. Independent samples *t*-test revealed that there was no difference in glucose concentrations between FAST and FED at each time period.

### Cellular damage biomarkers

Cellular damage biomarkers before and at the end of Ramadan are presented in Table 7. The two-way ANOVA (Ramadan × group) for CK, LDH, AST, ALT, γ-GT and PA concentrations revealed no significant effects for Ramadan, no significant effect for group or interaction between the two. Paired samples *t*-test revealed that CK, LDH, AST, ALT, γ-GT and PA concentrations did not change during the duration of the study in either group. Independent samples *t*-test showed no significant differences in these parameters between the two groups at any time period.

**Table 7:** Cellular damage biomarkers, immunological and inflammatory parameters before and at the end of Ramadan, M ± SD

**Group**				**Ramadan effect**			**Group effect**			**Ramadan × group effect**		
				***F(1,14)***	***P*-value**	**η**_***p***_^**2**^	***F(1,14)***	***P*-value**	**η**_***p***_^**2**^	***F(1,14)***	***P*-value**	**η**_***p***_^**2**^
CK (IU•l^-1^)	FAST	310 ± 83	300 ± 94	0.26	0.62	0.01	0.17	0.69	0.01	1.05	0.32	0.06
[CV = 4.7%] ^a^	FED	305.5 ± 81.71	336 ± 91									
LDH (IU•l^-1^)	FAST	283 ± 50	290.5 ± 60.2	0.01	0.91	0	0.2	0.66	0.01	1.05	0.32	0.06
[CV = 4.5%]	FED	277 ± 64	271 ± 68									
AST (IU•l^-1^)	FAST	26 ± 4.	28 ± 3	0.18	0.69	0.01	0.28	0.6	0.002	0.1	0.75	0.002
[CV = 4.8%]	FED	24 ± 5	27 ± 3									
ALT (IU•l^-1^)	FAST	20 ± 3	23 ± 5	0.42	0.53	0.002	0.18	0.69	0.001	1.58	0.56	0.003
[CV = 4.3%]	FED	22.5 ± 4.31	23 ± 4									
PA (IU•l^-1^)	FAST	128 ± 41	135 ± 34	1.69	0.21	0.1	0.13	0.91	0	0.06	0.81	0.003
[CV = 4%]	FED	124 ± 39	134 ± 27									
γ-GT (IU•l^-1^)	FAST	17 ± 3	19 ± 3	2.05	0.17	0.12	2.75	0.12	0.16	0.38	0.55	0.03
[CV = 3.8%]	FED	20 ± 4	21 ± 3									
Total leucocytes (10^9^•l^-1^)	FAST	6.41 ± 1.03	6.59 ± 1.18	1.37	0.26	0.02	0.12	0.73	0.04	0.04	0.84	0.004
[CV < 2%]	FED	6.8 ± 0.53	6.86 ± 0.87									
Neutrophils (10^9^•l^-1^)	FAST	3.42 ± 0.61	3.58 ± 0.78	0.01	0.89	0.001	1.97	0.11	0.01	1.18	0.29	0.003
[CV < 2%]	FED	3.53 ± 0.46	3.4 ± 0.51									
Lymphocytes (10^9^•l^-1^)	FAST	2.59 ± 0.58	2.67 ± 0.52	1.8	13	0.02	0.17	0.69	0..04	1.97	0.11	0.07
[CV < 2%]	FED	2.93 ± 0.2	3.14 ± 0.28									
Monocytes (10^9^•l^-1^)	FAST	0.31 ± 0.16	0.28 ± 0.16	0.78	0.39	0.06	0.88	0.36	0.04	0.14	0.71	0.008
[CV < 2%]	FED	0.29 ± 0.11	0.22 ± 0.13									
C-reactive protein (mg•l^-1^)	FAST	6.2 ± 0.9	6.1 ± 0.7	0.19	0.67	0.01	0.39	0.54	0.02	0.05	0.82	0.003
[CV = 4.5%]	FED	6.4 ± 0.9	6.3 ± 0.8									

Note: FAST = subjects training in a fasted state; FED = subjects training in a fed state; ^a^ = inter-assay coefficient of variance. CK = Creatine kinase, LDH = lactatedehydrogenase, ALT = alanine aminotransferase, AST = aspartate aminotransferase, AP = alkaline phosphatase, γ-GT = γ-glutamyl transferase. Before Ramadan (Bef-R) = 2 days before beginning the fast; end of Ramadan (End-R) = 29 days after beginning the fast.

### Immune and inflammatory markers

Immune and inflammatory markers before and at the end of Ramadan are shown in Table 7. There was no significant effect for Ramadan, no significant effect for group and no significant interaction on leukocyte counts, neutrophils, lymphocytes, monocytes and C-reactive protein. Paired samples *t*-test revealed that those parameters did not change during the duration of the study in either group. Independent samples *t*-test showed no significant differences in these parameters between the two groups at any time period.

## Discussion

The primary purpose of this study was to evaluate the effect of participation in Ramadan on body composition and circulating markers of renal function, immunity and inflammation in men, who continue to perform resistance training. A second aim was to determine whether training at night (in the acutely fed state) altered the impact of Ramadan compared to when training was undertaken during the day (in a fasted state). Our results showed, contrary to our hypothesis, that whether resistance training was conducted in a fed or fasted state, no significant effect on body mass or body composition of bodybuilders was revealed after four weeks. In addition, even though Ramadan fasting induced changes in urinary and some biochemical parameters, these changes were not different according to the state (fed vs fasted) in which training occurred.

Body mass and body composition did not change in either FAST or FED during Ramadan. Our results do not concur with the other published studies [4,27]. For example, Trabelsi et al. [2] demonstrated that fasted-state aerobic training resulted in a decrease in body mass as well as fat percent in physically active men. However, those changes were absent if an equivalent amount of aerobic exercise was performed in a fed state during Ramadan [2]. The discrepancy between that finding and the present study is likely due to a difference in the exercise regime; aerobic exercise will provide a better stimulus to induce fat oxidation than does resistance training. Notably, participation in Ramadan alone appears to improve the ability to utilize lipid during aerobic exercise [28], perhaps, providing an increased opportunity to reduce body fat stores if exercise is performed regularly during the fasting month. It appears that despite participation in Ramadan, lean body mass was maintained with no increase in body fat percentage. This may be largely because of the lack of change of training volume in this bodybuilder cohort. In addition, it is worth noting that energy and macronutrient intakes did not change during Ramadan and were consistent with the recommendation proposed by Slater and Phillips [29] for bodybuilders to induce hypertrophy. However, the use of a non-invasive method to measure changes in body composition (e.g., DEXA) in future studies of Ramadan is warranted to confirm this finding.

Urine specific gravity increased during Ramadan in both groups, which is consistent with some degree of dehydration [30], was previously observed with high intensity exercise training [31]. This state of dehydration has been previously attributed to a reduction of fluid intake [2,5,6]. It is likely our results can be similarly explained. However, in our previous work we have observed the urine specific gravity of subjects performing aerobic exercise before breaking the fast increasing during Ramadan, but absent in subjects practicing the equivalent amount of aerobic exercise after breaking the fast [2]. However, it is worth noting that our subjects had only about 4 hours to consume food or fluid after sunset on the day before the sample collection during Ramadan. It may well be that this was insufficient time to allow full hydration. Thus, our results concerning the hydration status of our subjects may be influenced independently of Ramadan. Markers of renal function showed a similar trend, increasing in both groups. Those findings were previously observed in subjects practicing aerobic exercise during Ramadan [2]. Sodium and chloride concentrations increased in both groups during Ramadan. A chronic state of mild dehydration in both groups may explain the abovementioned increase of serum electrolytes and renal function markers. Interestingly Ramadan fasting did not affect serum potassium concentrations in FED. Due to the dehydration and the elevations in serum sodium that occurred in FED, one might expect that increases in serum potassium concentrations would also be observed. However, a decrease in potassium intake may have offset any effects on serum potassium caused by dehydration [32].

HDL-C increased during Ramadan in FAST and FED, at variance with our previous work [2]. The rise in HDL-C was explained previously by change in body mass [2,33] or fat intakes [34]. However, in the present study, body mass did not change in either group while fat intakes increased only in FED. Thus, the rise of proportion of fat intakes during Ramadan can explain the increase in HDL-C in FED; although mechanisms by which fasting increases HDL-C in FAST remain unclear. Further investigation is needed to resolve this issue.

Whether Ramadan fasting affects cellular damage was also investigated in the present study. Serum CK, ALT, AST, ALT, AP and γ-GT were measured to assess the effect of Ramadan fasting on cellular damage biomarkers of bodybuilders. Ramadan fasting did not affect any of these variables and is in accordance with previous reports observing sedentary persons [35]. Nevertheless, to our knowledge, our study is the first to investigate the effect of Ramadan fasting on these parameters in men who undertake resistance training during Ramadan.

Serum C-reactive protein concentrations reflect the activity of cytokine-mediated inflammatory processes and are roughly proportional to the extent of tissue injury [36]. C-reactive protein did not change in either group and this perhaps could be explained by the lack of effect of Ramadan fasting on cellular damage biomarkers. Akin to previous studies in judokas [37], Ramadan had no impact on leukocyte count. Thus, in this context at least, continuation of resistance training whilst participation in Ramadan can be performed safely.

It is worth noting that effect sizes of the parameters measured in the current study were consistent but rather low. This, and the small number of participants my have resulted in type II error for some of the parameters measured. With this in mind, replication of the study with more participants during Ramadan would be difficult because of recruitment, but may result in further significant findings. Nevertheless, we have previously observed metabolic changes with participation in Ramadan with similar numbers of subjects [28].

## Conclusion

In conclusion, hypertrophic resistance training, unlike aerobic training, was not affected, at least in terms of body composition and markers of immune and inflammatory systems, when performed in a fed compared to a fasted state during Ramadan. However, resistance training performed during Ramadan was associated with an improved lipid profile and evidence of mild dehydration which may alters parameters indicative of renal function.

In terms of a practical application, trainers should educate bodybuilders on the importance of hydration during the nighttime in order to compensate for the dehydration that occurs during daytime within the month Ramadan. In addition the trainers should stress the importance of adopting a nutritional protocol similar to that of the normal non-fasting period.

## Abbreviations

FAST: Subjects training in a fasted state; FED: Subjects training in a fed state; Bef-R: Before Ramadan; End-R: End of Ramadan; BMI: Body mass index; BF%: Body fat percentage; LBM: Lean body mass; RM: Repetition maximum; RPE: Rating of perceived exertion; TG: Triglycerides; TC: Total cholesterol; HDL-C: High-density lipoprotein cholesterol; LDL-C: Low-density lipoprotein cholesterol; CK: Creatine kinase; LDH: Lactatedehydrogenase; ALT: Alanine aminotransferase; AST: Aspartate aminotransferase; AP: Alkaline phosphatase; γ-GT: γ-glutamyltransferase; a: Inter-assay coefficient of variance.

## Competing interest

The authors declare that they have no competing interests.

## Authors’ contributions

All authors have made substantive intellectual contributions towards conducting the study and preparing the manuscript for publication. TK, GZ, JK, KS, MRJ, HA and ZKM were responsible for the study design, coordination of the study, and oversight of data collection and analysis. SRS assisted in manuscript preparation and the revision of final manuscript. All authors read and approved of the final manuscript.
